# 18S-NemaBase: Curated 18S rRNA Database of Nematode Sequences

**DOI:** 10.2478/jofnem-2023-0006

**Published:** 2023-04-21

**Authors:** Kaitlin Gattoni, Eli M. S. Gendron, Rebeca Sandoval-Ruiz, Abigail Borgemeier, J. Parr McQueen, Rachel M. Shepherd, Dieter Slos, Thomas O. Powers, Dorota L. Porazinska

**Affiliations:** Department of Entomology and Nematology, University of Florida, FL 32611 United States of America; Department of Plant Pathology, University of Nebraska-Lincoln, NE 68588 United States of America; Plant Sciences Unit, Flanders Research Institute for Agriculture, Fisheries and Food (ILVO), Burg. Van Gansberghelaan 96, 9820 Merelbeke, Belgium

**Keywords:** biodiversity, database, ecology, metabarcoding, nematodes

## Abstract

Nematodes are the most abundant and diverse animals on the planet but lack representation in biodiversity research. This presents a problem for studying nematode diversity, particularly when molecular tools (i.e., barcoding and metabarcoding) rely on well-populated and curated reference databases, which are absent for nematodes. To improve molecular identification and the assessment of nematode diversity, we created and curated an 18S rRNA database specific to nematodes (18S-NemaBase) using sequences sourced from the most recent publicly available 18S rRNA SILVA v138 database. As part of the curation process, taxonomic strings were standardized to contain a fixed number of taxonomic ranks relevant to nematology and updated for the most recent accepted nematode classifications. In addition, apparent erroneous sequences were removed. To test the efficacy and accuracy of 18S-NemaBase, we compared it to an older but also curated SILVA v111 and the newest SILVA v138 by assigning taxonomies and analyzing the diversity of a nematode dataset from the Western Nebraska Sandhills. We showed that 18S-NemaBase provided more accurate taxonomic assignments and diversity assessments than either version of SILVA, with a much easier workflow and no need for manual corrections. Additionally, observed diversity further improved when 18S-NemaBase was supplemented with reference sequences from nematodes present in the study site. Although the 18S-NemaBase is a step in the right direction, a concerted effort to increase the number of high-quality, accessible, full-length nematode reference sequences is more important now than ever.

Biodiversity has been a topic at the forefront of ecology in recent decades due in part to global environmental changes, including climate warming, species invasions, and land conversion (Sutherland et al., 2012; Scheffers et al., 2016; Alberts et al., 2020) that threaten species with redistributions and extinctions (Bellard et al., 2012; Bellard et al., 2021). Studies of biodiversity have allowed for recognition of at-risk ecosystems and improved conservation strategies (Posa et al., 2011; Wintle et al., 2018). Both applied and basic research on these topics has primarily focused on macroscopic aboveground organisms such as plants and animals, while microscopic belowground biota, including microfauna, have received less attention. As direct and indirect connections between plants and all animals (aboveground and belowground) are important, the assessment of the total biodiversity within ecosystems is imperative (Colwell 1997; Bodelier 2011; Trevilline et al., 2019; Cameron et al., 2019). Unlike macrofauna, microfauna are difficult to study with the naked eye due to their small size and cryptic morphology, and hence require the use of advanced tools such as high-resolution microscopy and DNA metabarcoding (Bredtmann et al., 2017).

As one of the most abundant and diverse animals on the planet, nematodes are vital for ecosystem functioning (Hodda et al., 2009; van den Hoogen et al., 2020). Through their ubiquitous nature (De Mesel et al., 2004; Pascal et al., 2008; Heidemann et al., 2014; Majdi and Traunspurger 2015), diverse feeding habits (e.g., bacterial and fungal feeders, plant and animal parasites, omnivores, and predators), and positioning at various trophic levels, nematodes contribute to ecosystem functions such as primary productivity, decomposition, and overall nutrient cycling (Gerlach 1978; Bonaglia et al., 2014; Nascimento et al., 2012; Gebremikael et al., 2016; Schratzberger et al., 2019). In addition, plant and animal parasites can negatively impact agricultural production and human health. Precisely because of their diversity and roles in ecosystems, nematodes are well recognized as biological indicators of environmental change in terrestrial, marine, and freshwater ecosystems, including pollution, environmental disturbances, and climate warming (Hodda et al., 2009; Neher, 2010; Porazinska et al., 2021; Ottoni et al., 2022).

Traditionally, nematode identification has relied on the use of microscopy and analysis of morphological characteristics. However, this approach requires expertise and time, prohibiting the handling of the large number of samples necessary to study nematode diversity patterns and mechanisms at large scales. More importantly, although morphology has been considered the gold standard of nematode identification, it may be prone to subjectivity and errors, particularly because only <30,000 of the estimated ~1 – 10 million potential species have been described (Hodda, 2022).

While molecular barcoding using Sanger sequencing of rRNA and mitochondrial gene markers can be effective for identification of a few individual specimens from a small pool of species (e.g., Kiewnick et al., 2014; Pagan et al., 2015; Powers et al., 2021), this approach, like morphology, becomes inefficient and cost prohibitive as the diversity of nematodes and the number of analyzed samples increases (Porazinska et al., 2009; Geisen et al., 2018; Bubnoff 2008). A more recent solution to the limitations of low-throughput nematode identification has been offered by high-throughput nematode metabarcoding. One of the most important applications of this approach is its ability to rapidly detect and identify all nematode sequences present within a community across hundreds of samples. The 18S rRNA has been the most widely utilized DNA marker target, primarily focused on the hypervariable V4 – V8 regions (Ahmed et al., 2019; Herren et al., 2020; Müller et al., 2019; Porazinska et al., 2009, 2010; Sapkota and Nicolaisen 2015; Schenk et al., 2020; Sikder et al., 2020; Waeyenberge et al., 2019), with the V1 – V2 and V9 regions being used to a lesser degree (Müller et al., 2019; Porazinska et al., 2018; Waeyenberge et al., 2019; Schenk et al., 2019) (Fig. 1).

**Figure 1:**
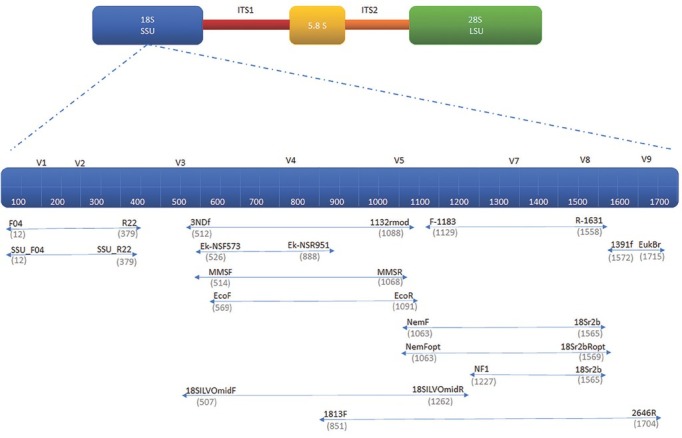
Genetic location of primers commonly used in nematode metabarcoding. Depicted is the entire rRNA gene with close ups of 18S rRNA gene with possible primers aligned below it. The 18S rRNA primers were aligned with *Caenorhabditis elegans* SSU (GenBank accession number: AY268117, X03680 and MN519140) to define the base pair locations (indicated in grey). References for primer sets available in Supplemental Table 1.

**Table 1:** The identities and number of 3 most populated genera across nematode orders in SILVA v111, SILVA V138, and 18S-NemaBase. The list is sorted from the largest to smallest number of total representative sequences.

	Number of Taxa			
Order	Genus	V111	V138	18S-NemaBase
Rhabditida	*Meloidogyne*	109	218	238
	*Caenorhabditis*	59	228	213
	*Bursaphelenchus*	77	116	117
Trichinellida	*Trichinella*	12	564	563
	*Capillaria*	1	16	16
	*Aonchotheca*	0	30	30
Dorylaimida	*Xiphinema*	148	137	137
	*Longidorus*	47	92	92
	*Enchodelus*	9	12	12
Enoplida	*Halalaimus*	43	43	43
	*Oxystomina*	26	26	26
	*Oncholaimus*	22	25	25
Triplonchida	*Paratrichodorus*	34	57	57
	*Trichodorus*	26	46	46
	*Tripyla*	21	30	30
Desmodorida	*Leptonemella*	1	19	19
	*Robbea*	10	9	9
	*Laxus*	4	9	9
Plectida	*Plectus*	15	24	24
	*Chronogaster*	5	7	7
	*Camacolaimus*	2	5	5
Monhysterida	*Eumonhystera*	3	11	11
	*Monhystera*	3	8	8
	*Daptonema*	8	10	10
Mononchida	*Mylonchulus*	27	27	27
	*Mononchus*	7	11	11
	*Clarkus*	6	6	6
Araeolaimida	*Sabatieria*	8	9	9
	*Axonolaimus*	4	4	4
	*Ascolaimus*	4	4	4
Mermithida	*Isomermis*	9	9	9
	*Mermis*	2	4	4
	*Pheromermis*	0	2	2
Desmodorida	*Desmoscolex*	1	2	2
	*Cyartonema*	1	1	1

Although nematode metabarcoding has expanded our understanding of nematode biodiversity (e.g., Porazinska et al., 2012; Geisen et al., 2018; Treonis et al., 2018; Santiago et al., 2021), the “identification” of nematodes from 18S metabarcoding datasets is still a challenge, because the task is directly proportional to the size and the quality of databases used to assign taxonomy to sequences (Zepeda et al., 2015). Unfortunately, the currently available 18S databases are severely underpopulated for nematodes (Macheriotou et al., 2019; Waeyenberge et al., 2019; Ahmed et al., 2019). For example, SILVA, the most popular database for nematodes (Quast et al., 2013; Yilmaz et al., 2014), contains only ~5,600 18S rRNA nematode sequences representing 2,734 species as of 2022, which is under 1% of the estimated 1 – 10 million nematode species (Hodda, 2022). Furthermore, out of these limited sequences, many are unverified (i.e., environmental and/or uncultured samples) or erroneous, characterized by unstandardized taxonomic strings, and classified using no longer accepted taxonomy (Fig. 2) (Waeyenberge et al., 2019), often resulting in an inability to recover the identity of queried sequences reliably and accurately.

**Figure 2:**
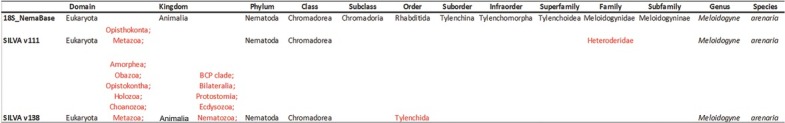
An example of variation of taxonomic categories and ranks resulting in variable strings for *Meloidogyne arenaria* across the databases. The curated 18S-NemaBase includes standardized taxonomic ranks consisting of 13 Nematoda relevant categories (top row) and currently accepted classification as adopted by the WoRMS database. In contrast, the outdated in-house curated SILVA v111 and the current v138 consisted of variable strings and/or outdated classification (indicated in red) and missing relevant to nematodes taxonomic information (blank cells).

Some of the above problems can be resolved through the access to a curated (curation being a process by which reference sequences are verified, organized, and standardized) reference database. For example, PR2, the 18S rRNA curated database for Protista, has significantly improved taxonomic assignments for this eukaryotic group (Guillou et al., 2013). Unfortunately, no up-to-date curated 18S database devoted specifically to nematodes is currently publicly available. Hence, our goal was to develop and provide an 18S rRNA curated nematode-specific database as a shared public resource to simplify workflow and improve the quality of nematode identification from 18S metabarcoding data. To accomplish this, we collated all nematode reference sequences from the most current SILVA v138, standardized their taxonomic strings, and updated classifications to be compliant with WoRMS (World Register of Marine Species) formatted taxonomy based on the Nemys repository (Nemys 2022; Vandepitte et al., 2018; WoRMS Editorial Board 2022). We also removed redundancies and errors. To illustrate the benefits, we used a small subset (the family of Tobrilidae) of the nematode metabarcoding dataset from the Western Nebraska Sandhills (Gattoni et al., 2022), and compared nematode identities across different database versions: 1) the curated but outdated SILVA v111 (~2,500 nematode sequences released in 2012), 2) the most recent version of SILVA v138 (~5,600 nematode sequences released in 2020), and either 3) our curated 18S-NemaBase (~5,300 nematode sequences) in two versions where the length of reference sequences was unaltered (full length 18S sequences) (18S-NemaBase) or 4) trimmed to the region defined by the NF1/18Sr2b primers (18S-NemaBase trimmed). We then expanded the database by adding 191 18S rRNA Sanger reference sequences for nematodes isolated from our Sandhills projects (18S-NemaBase-supplemented) to demonstrate that even small database expansions can make a significant difference. In addition, we created a tree from the 18S-NemaBase to support phylogenetic analyses. Finally, to allow for database personalization, we provided documented code to add or modify database content. All resources including the 18S-NemaBase, tree, alignment, and code are available at the Worms et al. website (http://www.WormsEtAl.com/databases) and GitHub (https://github.com/WormsEtAl/18SNemaBase)

## Materials and Methods

### 18S-NemaBase Curation

Two versions of the ARB-SILVA ribosomal RNA gene sequence database (Quast et al., 2012; Yilmaz et al., 2014) were used as base datasets for the development of 18S-NemaBase: the outdated but curated SILVA v111 (2,515 nematode sequences) and the most recently released SILVA v138 SSU Ref NR 99 (5,623 nematode sequences). Both versions were filtered to only include sequences labeled as nematodes. The taxonomic strings, sequences, and accession numbers were pulled from both files by using the bash ‘grep’ tool with ‘Nematoda’ set as the criterion for inclusion in the final file (for details of all mentioned functions and code see https://github.com/WormsEtAl/18SNemaBase The output was a list of accession numbers and the matching taxonomies and sequences.

### Taxonomic Standardization

To address the issues associated with taxonomic inconsistencies (e.g., variable and incomplete taxonomic strings and outdated classification) (Fig. 2), we used the WoRMS taxonomic database (Vandepitte et al., 2018; WoRMS Editorial Board, 2022) as a template for the use of 13 standardized taxonomic ranks (domain, kingdom, phylum, class, subclass, order; suborder, infraorder, superfamily, family, subfamily, genus, species) and currently accepted nematode classification as present in the Nemys repository (Bezerra et al., 2022; De Ley and Blaxter, 2004; Nemys, 2022). Full taxonomies across all ranks were pulled from the WoRMS database using a custom Python 3 script (taxonToFullTaxonomy.py modified from Sevigny’s code at https://github.com/Joseph7e/Nematode-Mitochondrial-Metagenomics/blob/main/correct_ncbi_based_on_worms.py).To apply these full standardized taxonomies to our 18S-NemaBase, we first pulled all nematode reference sequence taxonomic strings (along with accession numbers) using the standard bash ‘awk’ tool, tagged the genus and species ranks, and then matched the genus rank to the corresponding WoRMS’s taxonomy with a custom Python script. Corrected taxonomic strings were then manually double-checked for errors (see below). The updated strings were matched back to their reference sequences using the accession numbers with the bash ‘grep’ and ‘sed’ tools.

### Sequence Quality

To help eliminate redundancy, to reduce the overall computational and storage load of the database, and to identify potential errors, sequences were subjected to alignments and phylogenies. First, all sequences were grouped by subclass (i.e., Enoplia, Dorylaimia, and Chromadoria) and the Chromadoria were further grouped by orders (i.e., Araeolaimida, Chromadorida, Desmodorida, Desmoscolecida, Monhysterida, Plectida, and Rhabditida) using the ‘grep’ and ‘seqtk’ functions. Sequences were then aligned using the Muscle aligner (Edgar 2004), and Maximum-Likelihood trees using FastTree under a generalized time-reversible model were generated (Price et al., 2010). A custom dereplication bash script (extract_replicates_loop.sh) was used to identify any sequences that were deemed identical at a branch length of 0.0 threshold on the phylogenetic trees. Sequences that were deemed identical (i.e., 100% equivalent sequences and species identity) were further confirmed manually with Blast against the NCBI database to ensure the species and subspecies names were current. If multiple sequences provided a 100% match and were assigned to the exact same species/subspecies, only one was retained. However, if they matched different species/subspecies, both were retained. Sequences which were misplaced on trees or could not be confidently identified to the species level were deemed “poor-quality.” “Poor-quality” sequences were manually verified by examining their history, origin, and publication status using the NCBI database. Sequences that were unverified or incorrectly identified were removed.

After quality checking, MAFFT was used to align all the curated sequences of the 18S-NemaBase (Katoh and Standley 2013) and FastTree with default parameters was used to generate a Maximum-Likelihood tree as a reference for phylogenetic analyses. MAFFT was used for the alignment of all curated sequences instead of Muscle, as previously described, because it can better handle a large number of sequences. To allow for taxonomic assignments to sequences generated specifically by the NF1/18Sr2b primers, we also trimmed the alignment to the above barcoding region using MEGA v11 (Koichiro et al., 2021).

### Database Testing

To illustrate the potential benefits of the 18S-NemaBase curation on assigned nematode identity and diversity assessments, we used a small subset (the family of Tobrilidae) of the nematode metabarcoding dataset from the Western Nebraska Sandhills collected in 2019 and generated with NF1/18Sr2b primers (Gattoni et al., 2022). These nematodes reside within sediments of five lakes (Island, Gimlet, Bean, Kokjohn and Border Lakes) spanning an alkalinity gradient (pH 7-10). For all details of data generation and processing see Gattoni et al. (2022), but briefly demultiplexed sequencing data were processed with Qiime2 v2021.4 using cutadapt to remove primers (Martin, 2011) and DADA2 for sequence joining, filtering, and checking for chimeras (Callahan et al., 2016). To isolate nematode sequences from other taxa, we first assigned taxonomy to amplicon sequence variants (ASVs) with BLAST against our older curated but outdated SILVA v111 and removed all non-nematode sequences. In addition, any nematode ASVs with low numbers of reads (<5), low percent ID (<90%), and low query coverage (<99%) were removed. Because the presence of the “BCP clade” in SILVA v138 predictably resulted in truncated taxonomy and no hits to Nematoda, for the sake of analyses, two versions of SILVA v138 were produced: one containing “BCP clade” (thus referred to as SILVA v138-unmodified), and one with “BCP clade” manually corrected (thus referred to as SILVA v138-modified). We then used this SILVA v111 filtered nematode dataset to assign taxonomy against the following: 1. SILVA v138-unmodified, 2. SILVA v138-modified, 3. 18S-NemaBase, 4. 18S-NemaBase trimmed to NF1/18Sr2b amplicon, and 5. 18S-NemaBase-supplemented. The 18S-NemaBase-supplemented included 191 additional 18S rRNA Sanger reference sequences generated for a select group of nematode species present in our samples. The individuals of these species were extracted and identified morphologically via an inverted microscope followed by single nematode molecular DNA barcoding at the University of Nebraska as described by Powers and Harris (1993). Validated Sanger sequences (via taxonomic assignment statistic indices at NCBI and tree building as described in Powers et al., 2017) were then added to the 18S-NemaBase. This resulted in 6 ASV tables (1. SILVA v111, 2. SILVA v138-unmodified, 3. SILVA v138-modified, 4. 18S-NemaBase, 5. 18S-NemaBase trimmed to NF1/18Sr2b amplicon, and 6. 18S-NemaBase-supplemented).

## Results

### Database Comparison

SILVA v111 and v138 contained 2,515 and 5,623 nematode sequences respectively, constituting ~0.3% of all eukaryotic sequences in both databases. As part of the curation process, 209 “Nematoda” sequences that could not be identified in either family, genus, or species level were removed. Of the removed sequences, 89 were “uncultured_eukaryota,” “uncultured_microeukaryota,” or “uncultured_metazoan,” and 99 were assembled metagenome sequences labelled as “nematodes.” In addition, we identified and removed a total of 391 potentially erroneous sequences (e.g., extremely short sequences with equal hits to a wide variety of taxa or clearly non-nematode sequences). In result, our curated 18S-NemaBase included 5,232 nematode sequences all classified to at least the family level. The 5,232 sequences represent 14 orders, 214 families, 668 genera, and 2,734 species.

All taxonomic strings in both SILVA versions required standardization (Fig. 2). In SILVA v111, there were two uninformative classification categories for nematodes (i.e., Opisthokonta, Metazoa) and most taxonomic ranks were missing (i.e., kingdom, class, order, suborder, infraorder, superfamily) (Fig. 2). In SILVA v138, there were multiple uninformative classification categories (i.e., Amorphea, Obazoa, Opisthokonta, Holozoa, Choanozoa, Metazoa, BCP clade, Bilateria, Ecdysozoa, Nematozoa) but again, the most informative nematode ranks were missing (i.e., kingdom, class, order, suborder, infraorder, and superfamily). Additionally, the presence of the “space” character in the “BCP clade” predictably resulted in truncated, incomplete taxonomic strings, and required correction to retrieve nematode identities. To prevent these limitations, all nematode taxonomic strings in our 18S-NemaBase have been standardized to the strings modeled in WoRMS.

**Figure 3:**
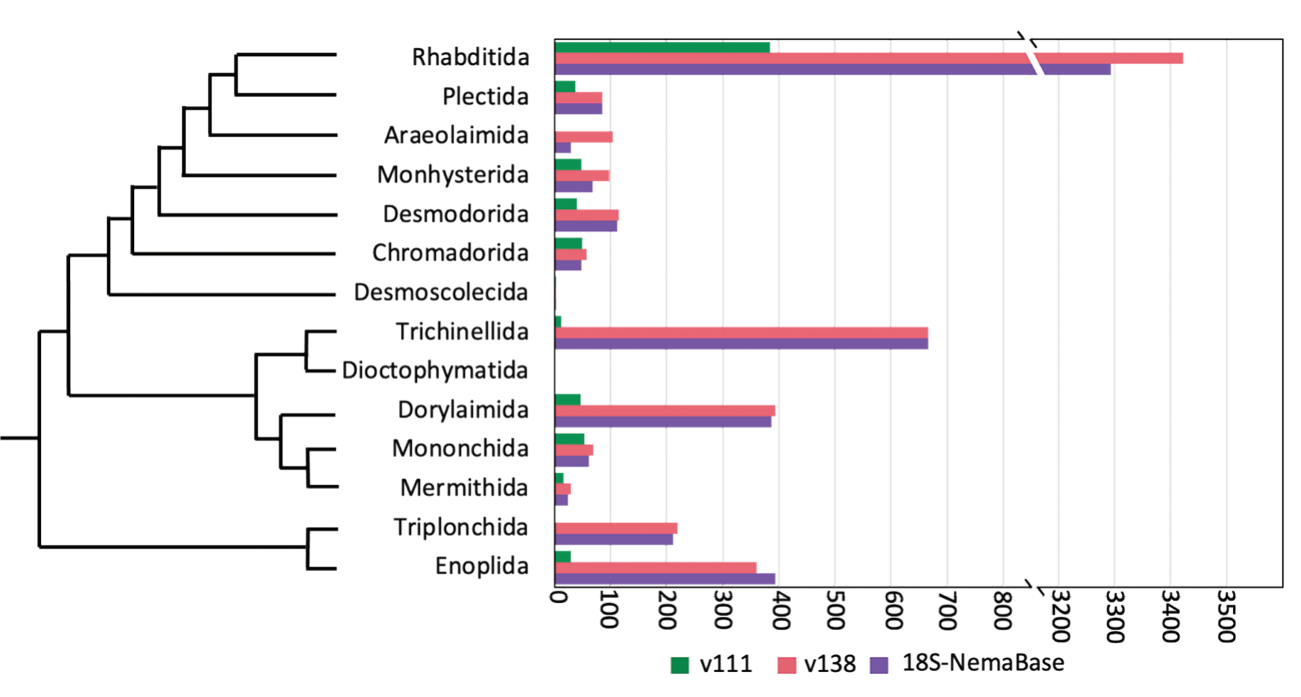
Number of available 18S reference sequences for Nematoda at the order level within SILVA v111, v138, and 18S-NemaBase.

Among 18S-NemaBase sequences, the majority belongs to Rhabditida (61%), followed by Trichinellida (12%), Dorylaimida (7%), Enoplida (7%), and Triplonchida (4%) (Fig. 3). A comparison between SILVA v111 and 18S-NemaBase indicates that the coverage for Rhabditida has increased 11-fold (384 vs. 3293 sequences, respectively) with the highest current representation of plant parasites (33.0%), followed by bacterial feeders (21.7%), animal parasites (22.1%), fungal feeders (14.0%), predators (4.6%) and root associates (4.6%) (Yeates et al., 1993). Additionally, the number of sequences for Triplonchida, Trichinellida, Enoplida, and Dorylaimida has increased 212-fold, 55-fold, 13-fold, and 8-fold, respectively. Overall, most orders experienced an increase of sequence representation, including Plectida, Araeolaimida, Monhysterida, Mononchida, and Mermithida, despite their general low coverage of <100 sequences per each clade in the 18S-NemaBase. Chromadorida was the only order that experienced the opposite pattern (2%), largely due to the removal of erroneous sequences and/or replacement of the outdated classification. Desmoscolecida and Dioctophymatida have been the most poorly represented orders, with only 3 and 2 sequences respectively.

The increase of sequence representation from SILVA v111 to 18S-NemaBase (Table 1) was particularly significant for animal and plant parasitic nematodes. For example, the number of sequences representative of *Trichinella* species increased from 12 to 563. There were many taxa, however, that did not observe any increase. For example, *Halalaimus*, the most common genus in Enoplida represented in the database, remained represented by 43 sequences in all versions of the databases.

The number of nematode sequences for most orders were similar between SILVA v138 and the new 18S-NemaBase. The largest discrepancy applied to Rhabditida, where SILVA v138 contained 129 more sequences than 18S-NemaBase. These sequences were removed during quality assessment using taxonomic trees and manual checking, as they were designated as uncultured, environmental, or erroneous.

### Effects on Metabarcoding Data

The 2019 Sandhills nematode ASVs assigned against 6 databases as described above were compared. The assignment from SILVA v138-unmodified without manually correcting for the “BCP clade” issue, resulted in not a single ASV assigned to “Nematoda” (Table 2). The assignments resulting from the 18S-NemaBase trimmed version were identical to the 18S-NemaBase non-trimmed full length sequence version; consequently, they are not discussed further. The 18S-NemaBase-supplemented contained an additional 191 unique Sanger sequences, of which 40 represented Tobrilidae, and as such, it is most relevant to the subset of our interest here.

The most distinguishing difference between the databases was associated with the number of undetermined identities, with 30 ASVs being assigned to “uncultured_nematode” and 16 ASVs to “Nematoda environmental samples” when using SILVA V111 and v138, respectively (Table 2). In contrast, when using 18S-NemaBase or 18S-NemaBase-supplemented, due to the curation process and removal of sequences with ambiguous identities, all ASVs were assigned to well-defined nematode taxonomies.

Moreover, we identified 18 ASVs (8% of total ASVs) with identities least defined by the SILVA v111 and best defined by the 18-NemaBase-supplemented. Out of these 18 ASVs, 13 belonged to the family Tobrilidae, a common nematode in aquatic systems and the most dominant component of our alkaline lakes. Out of the 13 ASVs, we identified 2 Tobrilidae species with SILVA v111, 4 with SILVA v138, 5 with 18S-NemaBase and 6 with 18S-NemaBase-supplemented with 4 matching Sandhill specific species (Table 2). In particular, three major species assigning to Sandhills specific nematodes comprised ~80% of the total Tobrilidae ASVs (Table 2). Most importantly, with the 18S-Nemabase, the time and effort to isolate/filter ASVs to taxa of specific interest (e.g., family of Tobrilidae), has been reduced to a matter of seconds.

**Table 2:** Tobrilidae species from the Western Nebraska Sandhills dataset assigned by v111, v138, and 18S-NemaBase, and 18S-NemaBase-supplemented databases. The numbers represent how many distinct ASVs assigned to that species.

Family	Genus	Species	v111	v138-unmodified	v138-modified	18S-NemaBase	18S-NemaBase-supplemented
Tobrilidae	*Brevitobrilus*	*Brevitobrilus* sp. Female SALCI Border	0	0	0	0	3
Tobrilidae	*Epitobrilus*	*Epitobrilus* sp. Male SALCI Border	0	0	0	0	4
Tobrilidae	*Epitobrilus*	*Epitobrilus stefanskii*	0	0	5	5	0
Tobrilidae	*Neotobrilus*	*Neotobrilus* sp. Female SALCI Island	0	0	0	0	1
Tobrilidae	*Semitobrilus*	*Semitobrilus* cf. *pellucidus* 1 JH-2014	0	0	3	3	0
Tobrilidae	*Tobrilus*	*Tobrilus* cf. *gracilis 2* JH-2014	0	0	1	1	0
Tobrilidae	*Tobrilus*	*Tobrilus gracilis*	6	0	0	0	0
Tobrilidae	*Tobrilus*	*Tobrilus pellucidus*	0	0	1	1	1
Tobrilidae	*Tobrilus*	*Tobrilus* sp. Female SALCI Island	0	0	0	0	1
Tobrilidae	*Tobrilus*	*Tobrilus* sp. ZQZ-2010a	*7*	0	0	3	3
		Total ASV/species	13/2	0	10/4	13/5	13/6
		Nematode_environmental sample	0	0	14	0	0
		Nematode_uncultured eukaryote	1	0	3	0	0
		Uncultured nematode	29	0	0	0	0
		BCP Clade	0	178	0	0	0

## Discussion

The accuracy and precision of 18S rRNA nematode metabarcoding is dependent on an up-to-date and well populated reference database. The current 18S rRNA database options for taxonomic assignments are inundated with multiple problems including incomplete taxonomies, outdated classifications, and erroneous/redundant sequences. We collated and curated a nematode-specific 18S rRNA reference database to overcome these problems and to improve the analysis of nematode diversity from metabarcoding data.

The lack of a curated reference database has been repeatedly cited as one of the major obstacles in nematode metabarcoding analysis (Powers et al., 2021; Schenk et al., 2020; Macheriotou et al., 2019; Waeyenberge et al., 2019). As the largest and most comprehensive 18S database, SILVA has been popular among nematologists and others studying bacterial and eukaryotic communities (Quast et al., 2013). While the newest v138 database contains almost three times the number of nematode sequences in comparison to the older v111, the taxonomic strings associated with v138 are incomplete and/or outdated. Additionally, v138 has the added issue associated with the “BCP clade” classification, resulting in truncated strings prior to the rank for “Nematoda” and in the potential inability to recover any sequence assignments of nematode origin. Although our curated SILVA v111 has provided some level of curation (e.g., length of taxonomic strings), it has become outdated both in terms of its underrepresentation and currently accepted nematode classifications. To address these problems, we created a curated database containing the most up to date 18S rRNA sequence collection for nematodes.

The updated database allows nematologists and other scientists studying nematode biodiversity to classify a broader range of diversity more accurately in at least three main ways which we demonstrated using our own samples from the Nebraska Sandhills. First, without any need for manual corrections, we easily retrieved nematode sequences (all 178 nematode ASVs). Second, with standardized taxonomic ranks and updated classification, we were able to expediently isolate the focal group of the enoplid Tobrilidae (13 ASVs representing >100,000 total reads). Finally, sequences assigned to “uncultured_ eukaryotes” with v138 (3 ASVs representing ~32,000 reads thus excluded from analyses) were reclassified to Tobrilidae with 18S-NemaBase. With more species recovered, 18S-NemaBase has allowed for a more precise understanding of Tobrilidae diversity in the Sandhill alkaline lakes compared to SILVA databases.

By adding custom Sanger sequences obtained directly from nematodes isolated from the Sandhills ecosystem, we further improved taxonomic assignments with 9 out of 13 total Tobrilidae ASVs representing ~87,000 reads reassigning to the custom sequences and species. These results illustrate that to make significant leaps in understanding of nematode diversity, there is a dire need for curated databases.

Equally important is the need for the work of taxonomist experts to expand 18S-NemaBase to a wider range of the nematode phylogenetic tree, feeding traits, ecosystems, and habitats. Our comparison of the coverage of taxa in the databases illustrates this need very well. For example, within the 8 years separating SILVA v111 and SILVA v138, many taxa remained underrepresented, including key plant parasites like *Xiphinema* and most of the Enoplida, the earliest branching order. As the most numerous and abundant group of multicellular animals on the planet, current nematode databases present a very shallow understanding of their distribution, diversity, and ecology. In the most current assessment of global nematode distribution, van den Hoogen et al. (2019) pointed out that our current knowledge of nematode abundance and distribution is largely limited to Europe and nematode taxa with clear or potential economic impact. Indeed, within the order Rhabditida, 55.1% of total nematode sequences in 18S-NemaBase represent plant- or animal-parasitic species. However, the bacterial-feeding rather than plant- or animal-parasitic nematodes have been estimated to be the most abundant globally (van den Hoogen et al., 2019). Additionally, the most well-represented bacterial-feeding taxon in our 18S-NemaBase belongs to *Caenorhabditis*, which includes *C. elegans*, a model nematode for evo-devo studies (Brenner 1974; Baker and Woolard 2019). This is problematic because its overrepresentation occludes the identification of other bacterial-feeding nematodes that play significant roles in ecosystem functioning including nutrient cycling and decomposition, thus remaining undescribed and uncharacterized (de Mesel et al., 2004; Heidemann et al., 2014; Majdi & Traunspurger, 2015; Pascal et al., 2008).

In conclusion, well-populated databases have been at the core of genomics since its beginning (Varmus 2002). To begin to alleviate some of the most notorious problems for nematode metabarcoding, we produced the 18S-NemaBase (all resources available at Wormsetal.com) and showed its benefits by improving on the assessment of the diversity of Tobrilidae from the Western Nebraska Sandhills. To continue to improve on nematode diversity analyses in the future, we need to make a concerted effort toward 18S-NemaBase expansion.

## Figures and Tables

**Supplemental Table 1:** List of primers depicted in Figure 1. Details include the primer name, author, and citation.

Primer	Author	Citation
F04 - R22	Fonseca et al., 2019	Fonseca, V. G., Carvalho, G. R., Sung, W., Johnson, H. F., Power, D. M., Neill, S. P., Packer, M., Blaxter, M. L., Lambshead, P. J. D., Thomas, W. K., and Creer, S. 2010. Second-generation environmental sequencing unmasks marine metazoan biodiversity. Nature Communications 1(98). http://https://dol/10.1038/ncomms1095
SSU_F04 - SSU_R22	Blaxter et al., 1998	Blaxter, M. L., De Ley, P., Garey, J. R., Liu, L. X., Scheldeman, P., Vierstraete, A., Vanfleteren, J. R., Mackey, L. Y., Dorris, M., Frisse, L. M., Vida, J. T., and Thomas, W. K. 1998. A molecular evolutionary framework for the phylum Nematoda. Nature 392:71-75. http://https://dol/10.1038/32160
3NDf- 1132rmod	Geisen et al., 2019	Geisen, S., Snoek, L. B., ten Hooven, F. C., Duyts, H., Kostenko, O., Bloem, J., Martens, H., Quist, C. W., Helder, J. A., and van den Putten, W. H. 2018. Integrating quantitative morphological and qualitative molecular methods to analyse soil nematode community responses to plant range expansion. Methods In Ecology and Evolution 9:1366-1378. http://https://dol/10.1111/2041 -210x.12999
Ek-NSF573 - EK-NSR951	Mangot et al., 2013	Mangot, J.-F., Domaizon, I., Taib, N., Marouni, N., Duffaud, E., Bronner, G., and Debroas, D. 2013. Short-term dynamics of diversity patterns: evidence of continual reassembly within lacustrine small eukaryotes. Environmental Microbiology 15: 1745-1758. http://https://dol.org/10.1111/1462-2920.12065
MMSF - MMSR	Sidker et al., 2020	Sikder, M. M., Vestergård, M., Sapkota, R., Kyndt, T., and Nicolaisen, M. 2020. Evaluation of Metabarcoding Primers for Analysis of Soil Nematode Communities. Diversity 12(388). http://https://dol.org/10.3390/d12100388
EcoF - EcoR	Waeyenberge et al., 2020	Waeyenberge, L., Sutter, N. D., Viaene, N., and Haegeman, A. 2019. New Insights Into nematode DNA-metabarcoding as revealed by the characterization of artificial and spiked nematode communities. Diversity 11 (4):52. http://https://dol.org/10.3390/d11040052
18SILVOmidF - 18SILVOmidR	Waeyenberge et al., 2020	Waeyenberge, L., Sutter, N. D., Viaene, N., and Haegeman, A. 2019. New Insights Into nematode DNA-metabarcoding as revealed by the characterization of artificial and spiked nematode communities. Diversity 11 (4):52. http://https://dol.org/10.3390/d11040052
1813F- 2646R	Holterman et al., 2006	Holterman M., van der Wurff A., van den Elsen S., van Megen H., Bongers T., Holovachov O., Bakker J., and Helder J., 2006. Phylum-wide analysis of SSU rDNA reveals deep phylogenetic relationships among nematodes and accelerated evolution toward crown clades. Molecular Biology and Evolution 23:1792-1800. http://https://doi/10.1093/molbev/msl044
NemF - 18Sr2b	Porazinska et al., 2009 and Sapkota et al., 2015	Porazinska, D. L, Giblin-Davis, R. M., Faller, L, Farmerie, W., Kanzaki, N., Morris, K., Powers, T. O., Tucker, A. E., Sung, W., and Thomas, W. K. 2009. Evaluating high-throughput sequencing as a method for metagenomic analysis of nematode diversity. Molecular Ecology Resoures 9:1439-50. http://https://doi.Org/10.1111/j.1755-0998.2009.02611; Sapkota, R., and Nicolaisen, M. 2015. High-throughput sequencing of nematode communities from total soil DNA extractions. BMC Ecology 12(15) http://https://doi.org/10.1186/s12898-014-0034-4
NemFopt - 18Sr2bopt	Waeyenberge et al., 2020	Waeyenberge, L., Sutter, N. D., Viaene, N., and Haegeman, A. 2019. New insights into nematode DNA-metabarcoding as revealed by the characterization of artificial and spiked nematode communities. Diversity 11 (4):52. http://https://dol.org/10.3390/d11040052
F-1183 - R-1631	Müller et al., 2019 and Starke et al., 2016	Müller, C. A., Pereira, L. D., Lopes, C., Cares, J., Borges, L. G. D., Giongo, A., Graeff-Teixeira, C., and Morassutti. 2019. Meiofaunal diversity In the Atlantic Forest soil: A quest for nematodes in a native reserve using eukaryotic metabarcoding analysis. Forest Ecology and Management 453. http://https://doi.Org/10.1016/j.foreco.2019.117591; Starke, R., Kermer, R., Ullmann-Zeunert, L., Baldwin, I. T., Seifert, J., Bastida, F., von Bergen, M., and Jehmlich, N. 2016. Bacteria dominate the short-term assimilation of plant-derived N in soil. Soil Biology and Biochemistry 96:30-38. http://https://doi.Org/10.1016/j.soilbio.2016.01.009
NF1 - 18Sr2b	Porazinska et al., 2009	Porazinska, D. L, Giblin-Davis, R. M., Faller, L, Farmerie, W., Kanzaki, N., Morris, K., Powers, T. O., Tucker, A. E., Sung, W., and Thomas, W. K. 2009. Evaluating high-throughput sequencing as a method for metagenomic analysis of nematode diversity. Molecular Ecology Resources 9:1439-50. http://https://doi.Org/10.1111/j.1755-0998.2009.02611
1391f- EukBr	Amaral-Zettler et al., 2009 and Caporaso et al., 2012	Amaral-Zettler, L. A., McCliment, E. A., Ducklow, H. W., and Huse, S. M. 2009. A method for studying protistan diversity using massively parallel sequencing of V9 hypervariable regions of small-subunit ribosomal RNA genes. PLoS One 4(7): 1 —9. http://https://doi.org/10.1371/journal.pone.0006372; Caporaso, J. G., Lauber, C. L., Walters, W. A., Berg-Lyons, D., Huntley, J., Fierer, N., Owens, S. M., Betley, J., Fraser, L, Bauer, M., Gormley, N., Gilbert, J. A., Smith, G., and Knight, R. 2012. Ultra-high-throughput microbial community analysis on the lllumina HiSeq and MiSeq platforms. ISME Journal 6(8):1621-1624. http://https://doi.Org/10.1038/ismej.2012.8
